# Enhancing Communication and Trust in Advance Care Planning for Older Adults with Sickle Cell Disease

**DOI:** 10.1093/geroni/igaf122.2613

**Published:** 2025-12-31

**Authors:** Tamara Poole, Rania Mohamed, Francyess Denis Oliva, Teagan Callaway, John Strouse, Charity Oyedeji

**Affiliations:** Duke University School of Nursing, Durham, North Carolina, United States; Duke University School of Medicine, Durham, North Carolina, United States; Duke University School of Medicine, Durham, North Carolina, United States; Duke University School of Medicine, Durham, North Carolina, United States; Duke University School of Medicine, Durham, North Carolina, United States; Duke University School of Medicine, Durham, North Carolina, United States

## Abstract

Despite advancements in treatments, life expectancy for sickle cell disease (SCD) remains 20 years shorter than people without SCD, making advance care planning (ACP) important. There is a gap in literature on ACP preferences of older adults with SCD. This study aims to describe ACP preferences for older adults with SCD. We interviewed 19 older adults with SCD (age ≥ 50). Interview questions addressed comfort discussing end-of-life care, nature of prior ACP discussions, and preferences for ACP. The data was analyzed using conventional content analysis. There were three themes. Theme one was importance of family involvement. Participants described preferences to have family present during ACP. Many preferred not leaving the burden of end-of-life decisions to family, while others preferred their family making end-of-life decisions. Participants stated preferences for their healthcare proxy (HCP), but the majority reported no HCP documentation. Theme two was early ACP and appropriate timing. Participants described discussing ACP while sick in the hospital as inappropriate. Due to the unpredictably of SCD, many stated that ACP should start in early adulthood or when they’re capable of understanding their options. Theme three was limited ACP communication. Participants reported limited communication or lack thereof about ACP preferences. Most participants were open to communication with a trusted provider they perceived to be knowledgeable of their SCD to provide information on or complete ACP documents. They preferred discussing ACP in clinic without rushing or interruptions. This highlights the importance of early family-centered ACP to honor the wishes of older adults with SCD.

